# High-Performance Lithium-Ion Storage of FeTiO_3_ with Morphology Adjustment and Niobium Doping

**DOI:** 10.3390/ma15196929

**Published:** 2022-10-06

**Authors:** Shenghao Li, Xiaohuan Wang, Zhiming Shi, Jun Wang, Guojun Ji, Xinba Yaer

**Affiliations:** 1 School of Materials Science and Engineering, Inner Mongolia University of Technology, No. 49 Aimin Street, Hohhot 010051, China; 2 School of Chemical Engineering, Inner Mongolia University of Technology, No. 49 Aimin Street, Hohhot 010051, China

**Keywords:** FeTiO_3_, niobium doping, microstructure, lithium storage performance

## Abstract

Ferrous titanate (FeTiO_3_) has a high theoretical capacity and physical and chemical properties stability, so it is a potential lithium anode material. In this study, FeTiO_3_ nanopowder and nanosheets were prepared by the sol–gel method and the hydrothermal method. In addition, niobium-ion doping was carried out, the radius of Nb close to Ti so the Nb can easily enter into the FeTiO_3_ lattice. Nb can provide more free electrons to improve the electrochemical performance. Then, the effects of the morphology and niobium doping on the microstructure and electrochemical properties of FeTiO_3_ were systematically studied. The results show that FeTiO_3_ nanosheets have a better lithium storage performance than nanopowders because of its high specific surface area. A certain amount of niobium doping can improve the electrochemical performance of FeTiO_3_. Finally, a 1 mol% niobium-doping FeTiO_3_ nanosheets (1Nb-FTO-S) electrode provided a higher specific capacity of 782.1 mAh g^−1^ at 50 mA g^−1^. After 200 cycles, the specific capacity of the 1Nb-FTO-S electrode remained at 509.6 mAh g^−1^. It is revealed that an increased specific surface area and ion doping are effective means to change the performance of lithium, and the proposed method looks promising for the design of other inorganic oxide electrode materials.

## 1. Introduction

Lithium-ion batteries (LIBs) are widely used in portable electronics, power supplies of electric vehicles, large-scale energy storage devices, and many other fields [1,2]. With the rapid development, high-requirements comprehensive performance LIBs are required for various uses. Therefore, the development of LIBs with a high-energy density, superior power density, and long cycle life has become the focus of the electrochemical energy storage field. The current related research mainly focuses on investigating electrode materials [3,4] and electrolytes [5,6]. In particular, anode materials are very important in the development of high-performance LIBs. Among these, titanium-based lithium-ion battery anode materials have attracted increasing attention due to their advantages in terms of a stable structure, high coulomb efficiency, and reliable charge/discharge platform, the most common of which are TiO_2_ and Li_4_Ti_5_O_12_. However, TiO_2_ mainly includes intrinsically poor ionic and electronic conductivity and a low theoretical capacity of 168–335 mAh g^−1^ [7]. Li_4_Ti_5_O_12_ has a low electron conductivity (10^−13^ S cm^−1^) and low lithium-ion diffusion coefficient (10^−13^~10^−9^ cm^2^ s^−1^) leading to the poor conductivity of materials and poor ion transport capacity, especially under the condition of a large current charge, and the discharge polarization reaction is strong and the capacity attenuation is fast. Therefore, these problems limit their wide application.

FeTiO_3_ (FTO) is a typical transition-metal titanate with a high theoretical capacity of 530 mAh g^−1^ and is environmentally friendly and low cost. FeTiO_3_ has attracted increasing attention in recent years as a battery material. Researchers have prepared FeTiO_3_ with various morphologies: nanoflowers provided a reversible capacity of 200 mAh g^−1^ at 50 mA g^−1^ after 50 cycles [8]; FeTiO_3_ nanoparticles provided an initial capacity of 358.8 mAh g^−1^ and a capacity of 100 mA g^−1^ after 200 cycles [9]; a 3D network FeTiO_3_/TiO_2_@C flexible fiber membrane delivered a reversible capacity of 205.4 mAh g^−1^ after 100 cycles at a current density of 300 mA g^−1^ [10]; and TiO_2_-FeTiO_3_@C porous showed a reversible capacity of 494.5 mAh g^−1^ after 150 cycles at a current density of 100 mA g^−1^ [11]. Obviously, the morphology of the electrode has a great influence on its electrochemical performance. However, the effect of a morphology change on the lithium storage performance of an electrode has not received much attention in this system. Beyond that, most researchers improve the electrochemical performance of the FTO as an electrode material by a composite method. It is well known that ion doping can change the physical and chemical properties of materials by changing the phase composition and microstructure. In this study, FeTiO_3_ electrodes with different morphologies were prepared to study the influence of morphology on the cyclability and stability of lithium-ion batteries. In addition, the microstructure of a FeTiO_3_ material is changed by simple ion doping, so as to improve its lithium storage performance. Obviously, from the microscopy of the electrode, it is of great significance to improve its electrochemical performance by regulating the microstructure, such as oxygen defects and the energy band structure, and it has great significance for the subsequent research and development of anode materials for lithium-ion batteries.

## 2. Materials and Methods

### 2.1. Materials Preparation

The sol-gel technique was used to prepare FeTiO_3_ nanopowders (FTO-P) with 1:1 Fe/Ti molar ratio. To this end, 10 mL tetrabutyl titanate (Ti(OC_4_H_9_)_4_) (Aladdin, Shanghai, China) was first slowly dropped into 20 mL anhydrous ethanol (Sinopharm, Beijing, China) followed by intense stirring for 10 min to yield Sample A. Next, 11.878 g ferric nitrate (Fe(NO_3_)_3_·9H_2_O) (Aladdin, Shanghai, China) was dissolved in 20 mL anhydrous ethanol to form Sample B. Afterward, Sample B was slowly added to Sample A under stirring for 10 min to yield Sample C. The masses of products obtained at molar ratios of niobium to titanium of 0, 1, 5, and 10 mol% were estimated to be 0, 0.0794, 0.397, and 0.794 g, respectively.

In the doping experiment, niobium chloride (Aladdin, Shanghai, China) was dissolved in anhydrous ethanol to form Solution D, which was added to Solution C under stirring for 10 min. Next, ammonia solution (Sinopharm, Beijing, China) was added dropwise until the formation of a gel followed by aging for 24 h before use. Amorphous FeTiO_3_ powder was obtained by heating the aged sol at 80 °C for 24 h. Next, the dried gel was sintered at 600 °C for 2 h under mixed CO and Ar-reducing sintering atmosphere at respective fluxes of 100 and 400 mL/min, a heating rate of 5 °C/min, and cooling in the furnace after formation.

The hydrothermal method was used for the synthesis of FeTiO_3_ nanosheets (FTO-S). To this end, FeTiO_3_ nanopowder (0.1 g) was added into 30 mL NaOH (10 mol/L, Sinopharm, Beijing, China). The mixture was then placed in a stainless steel reactor (Boke, Zhengzhou, China) and heated at 100 °C for 12 h. The resulting sample was collected and cleaned with deionized water and vacuum dried at 60 °C. The obtained Nb-doped FeTiO_3_ nanosheets were denoted as 1Nb-FTO-S, 5Nb-FTO-S, and 10Nb-FTO-S, respectively.

The electrical properties of the materials were tested using a button battery (Canrd, Guangdong, China) as the carrier. The CR2032 button battery was composed of the positive electrode, negative electrode, diaphragm, electrolyte, shrapnel, gasket, shell, and part. The LIBs anode sheets were prepared by mixing acetylene black (Saibo, Beijing, China) and sodium alginate (Aladdin, Shanghai, China) at a mass ratio of 8:1:1 followed by grinding. Deionized water was then added to produce slurries of materials. After uniform grinding, each slurry was uniformly coated on the copper box through the scraper coating machine, and then dried in an oven at 80 °C for 12 h. Subsequently, electrodes with 14 mm diameter were prepared by slicing. The electrolyte was made of a 1 mol/L LIPF_6_ vinyl carbonate (EC) diethyl carbonate (DEC) mixture (Aladdin, Shanghai, China) at a volume ratio of 1:1. Polypropylene (PP) microporous membrane (Aladdin, Shanghai, China) was employed as membrane, and lithium metal sheet (diameter 12 mm, thickness 0.5 mm, Canrd, Guangdong, China) as the counter electrode. Due to facile oxidation and metamorphization, type 2032 button batteries were assembled in an argon-protected glove box.

### 2.2. Characterization

The crystal phases of the materials were identified by X-ray diffraction (XRD, D/MAX 2500 PC, Rigaku, Tokyo, Japan) with Cu Kα doublet radiation. Rietveld refinements of XRD patterns for doped FeTiO_3_ were accomplished by GSAS-EXPGUI program package [12]. The microstructure of FeTiO_3_ was characterized by field-emission scanning electron microscopy (SEM, SU8010, Hitachi, Tokyo, Japan). FeTiO_3_ powder was characterized by transmission electron microscopy (TEM) and the high-resolution TEM (HRTEM) (Tecnai G2 F20, FEI, Hillsboro, OR, USA). The chemical compositions of the samples were acquired by energy dispersive spectrometry (EDS, FEI, Hillsboro, OR, USA). X-ray photoelectron spectroscopy (XPS, ESCALAB 250Xi, Thermo Fisher Scientific, Waltham, MA, USA) was used to analyze the surface elemental composition, the valence state of the elements, valence band spectra (VBXPS), and reflected electron energy loss spectra (REELS). To further analyze the oxygen vacancies and Ti^3+^ information, the samples were tested by Bruker A300-10/12 electron paramagnetic resonance (EPR, Bruker, Karlsruhe, Germany) at 77 K, microwave frequency of 9.852 GHz, and field modulation frequency of 100 kHz.

The galvanostatic charge/discharge profiles of the electrodes were tested by LAND battery system (LANHE, Wuhan, China) at voltages of 0.005–3 V. To further investigate the electrochemical kinetics of the as-prepared electrodes, cyclic voltammetry (CV) and electrochemical impedance spectroscopy (EIS) were carried out. CV at voltages of 0.005–3 V, as well as EIS at an open-circuit voltage and frequencies of 0.01–10 MHz were obtained on a CH Instruments 660E electrochemical workstation (CH Instruments, Shanghai, China).

## 3. Results

### 3.1. Microstructure of Nb-Doping FeTiO_3_

**Figure 1:**
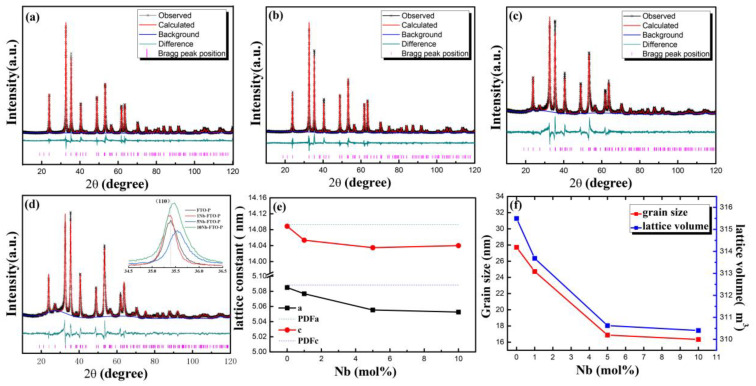
XRD patterns of Nb-doped FeTiO_3_ sintered at 600 °C. (**a**) FTO-P; (**b**) 1Nb-FTO-P; (**c**) 5Nb-FTO-P; (**d**) 10Nb-FTO-P and expanded view of (110) peak (inset); (**e**) lattice constant; (**f**) grain size.

The XRD Reitveld refinements of the Nb-doped FeTiO_3_ samples under a reducing atmosphere are shown in Figure 1a–d. All the diffraction peaks of the FTO-P and 1Nb-FTO-P were conformed to the Hexagonal FeTiO_3_ structure (PDF#29-0733) with the space group R-3 (148). In addition, no other phases were observed under the detection limit of the XRD machine, indicating that all Nb ions entered the lattice of the FeTiO_3_. However, the increase in the doping amount of Nb to 5 mol% revealed the presence of small amounts of Tetragonal TiO_2_ (PDF#21-1276) with the space group P42/mnm (136) in the samples besides the FeTiO_3_ phase, which is marked with a star in Figure 1c,d. After the structural refinement, the proportions of the FeTiO_3_ and rutile were estimated to be 90.2 and 9.8%, respectively. As the doping amount increased to 10 mol%, the FeTiO_3_ reached 88.9%, the rutile accounted for 11.0%, and the TiO_2_ phase increased slightly. Thus, Nb doping promoted the generation of TiO_2_. The reason for this had to do with the increase in Nb doping, which enhanced the internal energy of the FeTiO_3_ gradually while declining the stability of the FeTiO_3_ [13]. This, in turn, led to larger amounts of doping and the formation of new phases. Table 1 shows the structural parameters, including the atomic coordinates, occupancies, and the reliability factors.

In addition, the rise in the Nb-doping amount led to an obvious diffraction peak width in the XRD spectra because the doping resulted in distorted crystals. With the increase in the niobium-doping amount, the diffraction peak of (110) is obviously shifted to the high-angle direction (insert in Figure 1d), which also indicates that niobium doping enters the FeTiO_3_ lattice. The changes in the lattice constant, grain size, and cell volume of the Nb-doping FeTiO_3_ are depicted in Figure 1e,f. The lattice constant decreased relatively with the increase in the Nb-doping content, and both the grain size and cell volume also declined. The reason is that Nb doping introduced some defects, hindering further growth of the grain, then refining the FeTiO_3_ grain.

**Figure 2:**
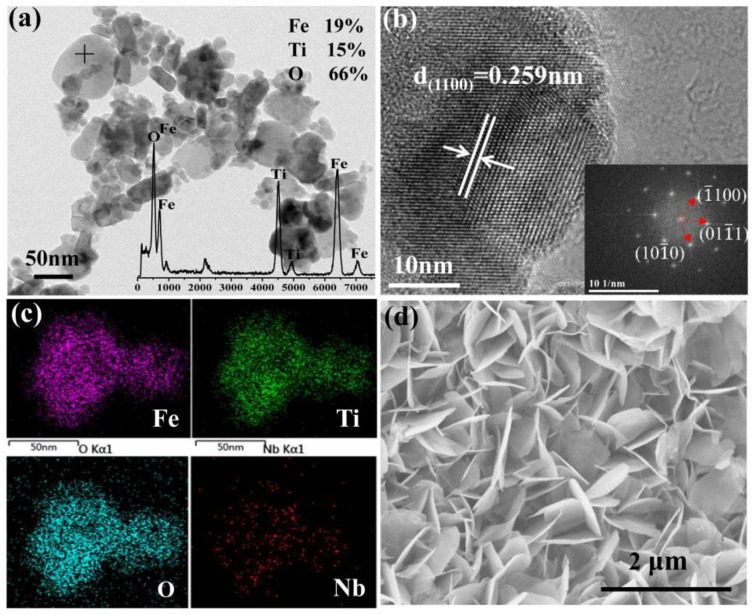
(**a**) TEM; (**b**) HRTEM; (**c**) EDS mapping of 1Nb-FTO-P; (**d**) SEM of 1Nb-FTO-S.

The TEM, HRTEM, EDS of 1Nb-FTO-P, and SEM of 1Nb-FTO-S are provided in Figure 2. For the 1Nb-FTO-P, the samples are nanoparticles, about 20–50 nm (Figure 2a), and the EDS inset shows the atomic ratios of Fe, Ti, and O were estimated to be 19, 15, and 66%, respectively. The high-resolution TEM in Figure 2b revealed grains with visible lattice stripes, in which the lower right corner was embedded in the right part of the selection diffraction spots obtained by HRTEM via Fourier transform. The interplanar crystal spacing was measured as d = 0.251 nm. The binding XRD analyses showed the FeTiO_3_ crystal diffraction peak corresponding to the FeTiO_3_ (110) crystal face. The diffraction spots are calibrated as the crystal planes of ($\bar{1}$100), (01$\bar{1}$0), and (10$\bar{1}$0) with a hexagonal structure of FeTiO_3_, which belong to the [0001] crystal belt axis. The EDS mapping images of 1Nb-FTO-P are shown in Figure 2c. The Nb entered the FeTiO_3_ lattice to form an even distribution after doping. The atomic ratios of Fe, Ti, and O were estimated to be 18, 19, and 63%, respectively. Hence, the Fe, Ti, and O substantial ratio was close to 1:1:3, satisfying the compound ratio of the determined chemical composition. The proportion of Nb was recorded as 0.95%, close to the added 1 mol% in the synthesis, once again confirming the penetration of bismuth into the FeTiO_3_ lattice. In the SEM imaging (Figure 2d), the sample presented a “petals” sheet shape.

**Figure 3:**
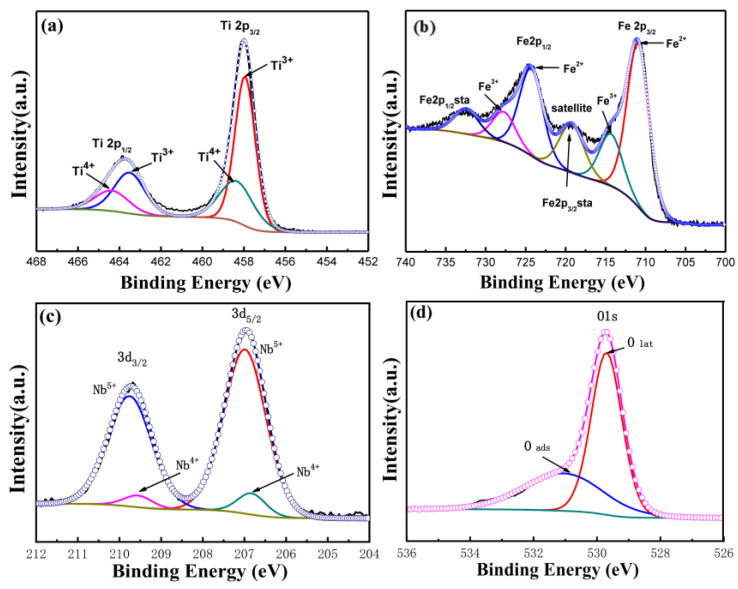
XPS spectra of 1Nb-FTO-S. (**a**) Ti2p; (**b**) Fe2p; (**c**) Nb3d; (**d**) O1s.

**Figure 4:**
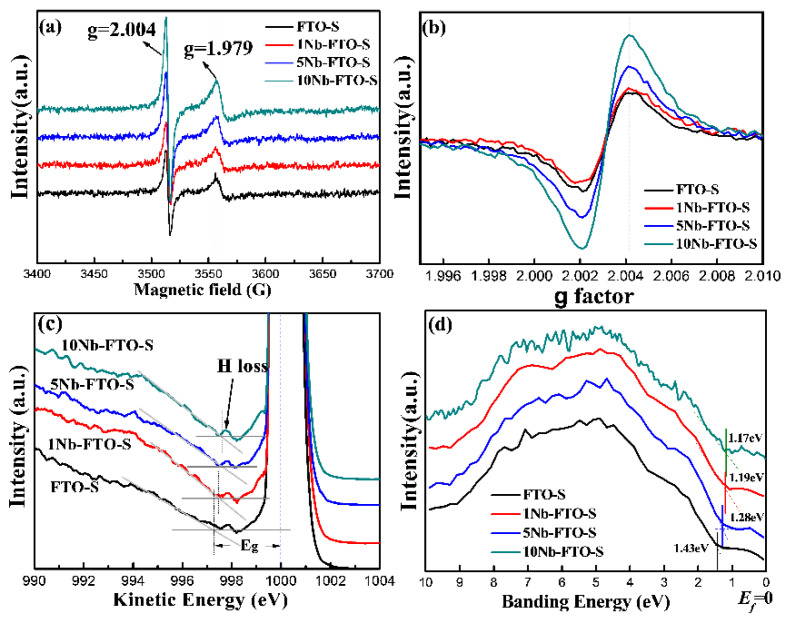
(**a**,**b**) EPR spectra; (**c**) REELS; (**d**) VBXPS spectra of different Nb-doped FeTiO_3_.

Figure 3 shows the XPS pattern of the 1Nb-FTO-S sample. The peak of the Ti2p was observed at binding energies of 457.9 and 458.3 eV, corresponding to Ti^3+^(2p_3/2_) and Ti^4+^(2p_3/2_), respectively. The binding energies at 463.5 and 464.4 eV were assigned to Ti^3+^(2p_1/2_) and Ti^4+^(2p_1/2_), respectively. As shown by the peak area, most of the Ti still existed in the form of Ti^3+^, while only small amounts of Ti^4+^ were present. The existence of more Ti^3+^ was attributed to calcination under the reducing atmosphere. The characteristic peaks of the Fe2p orbit are provided in Figure 3b. The Fe displayed characteristic peaks of Fe(2p_1/2_) and Fe(2p_3/2_), with binding energies differed by 13.6 eV [14], as well as a satellite peak with an Fe2p orbit between Fe(2p_1/2_) and Fe(2p_3/2_) [15,16]. Meanwhile, Fe(2p_3/2_) showed an obvious “shoulder peak”, indicating no single-valence state of Fe. The peak separation fitting suggested Fe coexisting in the form of bivalent and trivalent. The existence of satellite peaks was caused by phenomena such as electron relaxation, multiple splitting, and multi-electron effects. The Nb(3d_3/2_) and Nb(3d_5/2_) characteristic peaks of the Nb3d orbit are illustrated in Figure 3c. Small amounts of Nb existed as Nb^4+^, while more existed in the form of Nb^5+^. Figure 4d shows the characteristic peak of the O1s orbit. The characteristic peak corresponding to the low binding energy was assigned to the surface lattice oxygen (O_lat_), and that at a high binding energy was attributed to adsorbed oxygen in the lattice (O_ads_).

To gain more details about the defects in the Nb-doped FeTiO_3_ samples, the FTO-S, 1Nb-FTO-S, 5Nb-FTO-S, and 10Nb-FTO-S samples were characterized by EPR. Figure 4a,b show the EPR maps of FeTiO_3_ doped with different Nb concentrations. All samples revealed a strong isotropic resonance signal (Figure 4a) at the position g = 2.004, corresponding to a magnetic field intensity of 3512.91 g. This signal was attributed to O^2−^ free radicals [17,18], ultimately formed by the adsorption of air oxygen by surface oxygen vacancies. Therefore, the signal also belonged to the oxygen vacancies signal [18,19]. All the samples also showed a strong resonance signal at position g = 1.97949, corresponding to the center of the paramagnetic Ti^3+^ [20,21]. The amplified EPR spectrum is depicted in Figure 4b. Oxygen vacancies existed in the FTO-S sample. In addition, the intensity of the oxygen vacancy signal in the 1Nb-FTO-S sample was slightly lower than that in the FTO-S sample. As the doping Nb amount rose, the signal of the oxygen vacancies became gradually stronger, indicating gradually enhanced concentrations of the oxygen vacancies. In addition, the resonance signal strength of Ti^3+^ slightly incremented after Nb doping because high-price Nb^5+^ or Nb^4+^ doping into FeTiO_3_ would more likely form Ti^3+^ defects for charge compensation [22]. Because the prepared ferrous titanate powder was black, the band gap could not be measured by optical spectroscopy. The data revealed that the electron transition of the first excited state of the material can be inferred using the Reels technique. Some studies dealing with semiconductors or insulators indicated that the band gap can be determined by exciting the valence electrons to the conduction band [23,24,25]. Figure 4c shows the Reels spectra of the FTO-S, 1Nb-FTO-S, 5Nb-FTO-S, and 10Nb-FTO-S samples. A large peak with a kinetic energy of about 1000 eV was noticed in the elastic region. This strongest peak corresponded to the elastic peak when the electrons collided with other atoms except for H. A small peak existed in the direction of low kinetic energy corresponding to the value of kinetic energy caused by inelastic collisions between electrons and small numbers of H atoms on the sample surface [25]. The band gap (Eg) widths of the FTO-S, 1Nb-FTO-S, 5Nb-FTO-S, and 10Nb-FTO-S samples measured by the extrapolation method were determined as 2.70, 2.49, 2.41, and 2.38 eV, respectively. Note that this was the first time that the band structure information of FeTiO_3_ was analyzed by Reels, and the results were basically consistent with those reported in the literature [26,27,28]. The Reels results showed a gradual narrowing of the band gap with the increase in the Nb-doping amount, as the Nb doping may have introduced donor levels below the conduction band [29].

The influence of Nb doping on the band structure of FeTiO_3_ was further investigated by XPS to test the valence band spectral information. Note that the XPS valence band spectrum (VBXPS) could be used to qualitatively study the relative height of the top positions of the valence bands in semiconductor materials. In this respect, the highest energy position that can be filled by valence electrons is called the Valence Band Maximum (VBM). The band structures of the samples were analyzed by VBXPS, and the valence band spectra of the FTO-S, 1Nb-FTO-S, 5Nb-FTO-S, and 10Nb-FTO-S samples are shown in Figure 4d. The position of binding energy 0 eV was set as the Fermi level E_f_, and the position of the valence band top was set as the tangent line made by linear extrapolation along the abrupt ascending section of the initial edge of the valence band spectrum. The intersection point between the tangent line and horizontal line of the base noise was then recorded and used for the data analysis [23]. The top positions of the valence bands of the FTO-S, 1Nb-FTO-S, 5Nb-FTO-S, and 10Nb-FTO-S samples were estimated to be about −1.43, −1.28, −1.19, and −1.17 eV, respectively. The Fermi levels of the samples can be considered as the guide band movement, indicating an increase in the free-electron concentration in the guide band, as well as a decrease in the guide band, consistent with the above Reels data. On the other hand, the corresponding band gap widths of the FTO-S, 1Nb-FTO-S, 5Nb-FTO-S, and 10Nb-FTO-S samples measured by Reels were estimated to be 2.70, 2.49, 2.41, and 2.38 eV, respectively. The positions of the low conduction bands also changed by 1.27, 1.21, 1.22, and 1.21 eV, respectively.

### 3.2. Electrochemical Properties of FeTiO_3_ Samples

**Figure 5:**
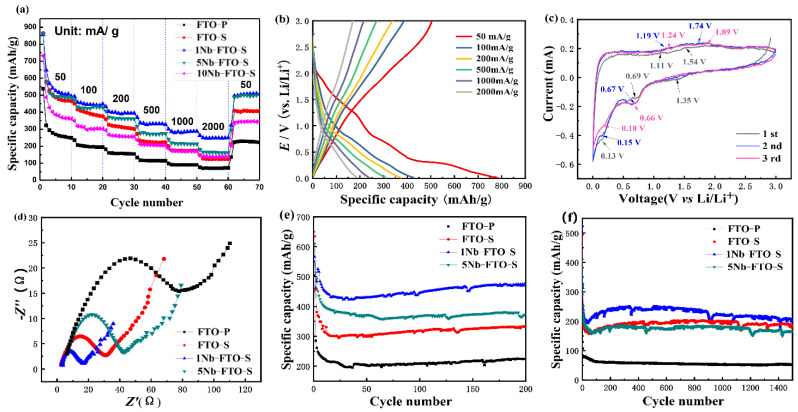
The electrochemical properties of Nb-doped FeTiO_3_ electrodes. (**a**) Rate capability; (**b**) charge/discharge curves; (**c**) CV curves; (**d**) EIS spectra; (**e**,**f**) cycling performance.

The electrochemical properties of the Nb-doped FeTiO_3_ electrodes for lithium storage are shown in Figure 5. The multiplier performance diagrams of the FTO-P, FTO-S, 1Nb-FTO-S, 5Nb-FTO-S, and 10Nb-FTO-S samples are provided in Figure 5a. Note that each sample was cycled 10 times at the same rate. The corresponding initial discharge capacities were recorded as 537.3, 863.8, 856.7, 865.5, and 732.7 mAh g^−1^, respectively.

The stable capacity of the FTO-P sample at the low current density of 50 mA g^−1^ was only about 250–270 mAh g^−1^, and the capacity decay increased rapidly with magnification. Meanwhile, the nanosheets (FTO-S) showed higher specific capacities than the nanopowder (FTO-P). After 10 cycles, the specific capacities of the FTO-S samples were all around 550 mAh g^−1^, a value much higher than that of the FTO samples. The reason for the improved rate performance of the nanosheets was linked to their morphologies and large specific surface areas. Our previous studies [29] have shown that the BET surface areas of FTO-P and FTO-S were estimated to be 40.77 and 86.28 m^2^ g^−1^, the pore volumes were 0.1 and 0.27 cm^2^ g^−1^, and the pore diameters were 9.84 and 11.02 nm, respectively. These results would be conducive to the rapid removal/fixation of lithium ions, providing more ions and electrons in the transport channels. This would also shorten the distance between the lithium ions and electrons, as well as effectively alleviating the volume change in the material during charge/discharge processes [30]. On the other hand, the sheet structure can also reduce the interface energy between the electrode and electrolyte in contact with the interface [31], so that the electrode reaction polarization became weakened to effectively improve the performance of the electrode material of LIBs [32].

For the Nb-doped samples, the specific capacity gradually attenuated with magnification. The latter was alleviated by small amounts of Nb-doped samples. Among the specimens, 1Nb-FTO-S (1 mol% Nb) exhibited a high reversible capacity of 856.7 mAh g^−1^ at 50 mA g^−1^. At 2000 mA g^−1^, the capacity can still reach about 250 mAh g^−1^, a value better than those of many titanium-based electrode materials at higher rates [7,20,33]. When it goes back to 50 mA g^−1^, the reversible capacities returned smoothly to the initial values of about 508.8 mAh g^−1^. Thus, the prepared electrode materials displayed an excellent rate performance and good cycle stability. However, the specific capacity of 5Nb-FTO-S (5 mol% Nb) and 10Nb-FTO-S (10 mol% Nb) looked relatively low with about 180 and 140 mAh g^−1^, respectively, at a current density of 2000 mA g^−1^. Meanwhile, the capacity decayed rapidly with the increase in magnification.

It can be clearly seen that only a small amount of Nb doping could slow down the attenuation of the electrode capacity. For the first reason, the XRD results prove that Nb doping led to refining the grain size, which played a decisive role in the diffusion time of the Li^+^ ions in the particles. Smaller grains would lead to shorter diffusion times of the Li^+^ ions and improve the performance of the LIBs. The second reason is that Nb doping introduces more Ti^3+^; the Ti^3+^/Ti^4+^ mixture acted as the charge compensation and the enhanced electron concentration, and the existence of Ti^3+^ also improved the overall electronic conductivity of the materials, thereby promoting their properties [30].

In addition, conductivity affects the electrode materials of LIBs, where better conductivity would lead to a smaller capacity loss and an improved ion transport capacity. Table 2 lists the conductivity of the Nb-doped samples. The conductivity of FTO-S was measured as 2.1 × 10^−3^ S cm^−1^, and that of 1Nb-FTO-S (1 mol% Nb) increased to 8.3 × 10^−2^ S cm^−1^, while the conductivity declined for the 5Nb-FTO-S (5 mol% Nb) and 10Nb-FTO-S (10 mol% Nb). Therefore, only a small amount of Nb doping could improve the lithium storage performance by enhancing the electrical conductivity. Nb doping introduced excess electrons, conducive to increased electrical conductivity. However, excessive niobium doping can produce TiO_2_ and may reduce the conductivity. Meanwhile, the energy level at the bottom of the conduction band moved toward lower energies, reducing the band gap width of the material as proved by the Reels results. As it was reported that narrow band gaps corresponding to faster electron transport kinetics [34].

**Figure 6:**
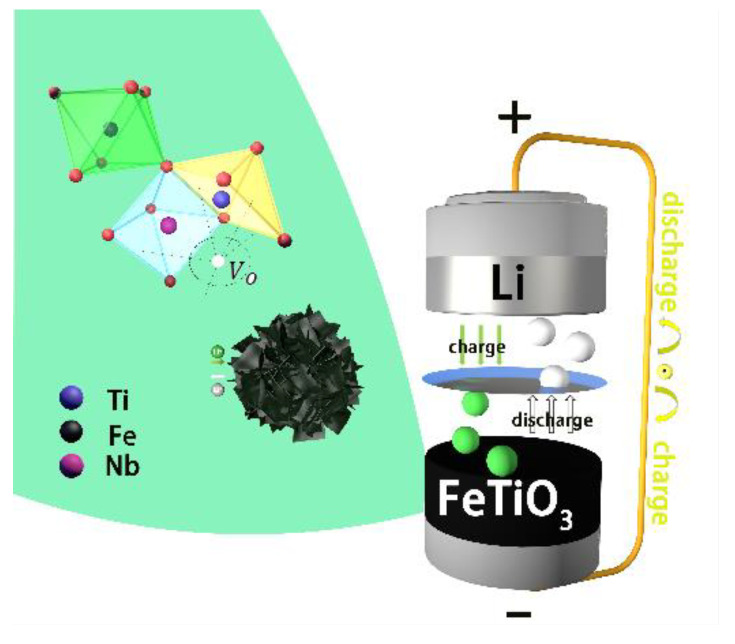
Mechanism diagram of lithium storage performance.

Furthermore, the existence of oxygen vacancies also exerted a certain effect on the performances of the LIBs electrodes. The interfacial charge transfer and reaction kinetics were both promoted by the interaction between the oxygen vacancies and electrons, improving the electrical conductivity [35,36]. The preliminary XPS and EPR analyses showed increased oxygen vacancy concentrations in each sample with the Nb-doping amount. However, an excessive oxygen vacancy will cause the diffusion migration of other ions in the electrode material, which will eventually lead to the degradation of the electrode performance [37], which may be one of the reasons for the deterioration of the battery rate and cycling performance due to high Nb doping (5 and 10 mol%). It can be seen that the existence of oxygen vacancies has a great influence on the physical and chemical properties of lithium-ion batteries. The mechanism diagram of the lithium storage performance is shown in Figure 6.

In conclusion, the morphology, valence state composition, conductivity, oxygen vacancy, and band structure of the sample together affect the electrochemical performance of the electrode, and 1Nb-FTO-S (1 mol% Nb) has the best rate performance and cycling performance due to its flake structure, certain microstructure, and high conductivity. However, the performance of excessive niobium-doped samples 5Nb-FTO-S and 10Nb-FTO-S deteriorating may be because of the occurrence of the TiO_2_ phase in the sample, which reduces the conductivity. In addition, the lattice structure of ferrous titanate became distorted at a very high Nb doping (5 and 10 mol%), leading to the resistance to the transfer of lithium ions, and eventually the performance deterioration [36].

Because a high rate capability was also required for practical applications, the rate performance of the 1Nb-FTO-S (1 mol% Nb) electrode was explored at different current densities. Figure 5b shows the charge–discharge curves of the 1Nb-FTO-S electrode at voltages of 0–3 V vs. Li^+^/Li and current densities of 50–2000 mA g^−^^1^. The sample illustrated no obvious discharge platforms. The first charge and discharge curves of the sample showed a large irreversible capacity, which may be due to the large specific surface area of the raw material, as well as a surface prone to the easy absorption of H_2_O/OH groups. As the current density increased, the specific capacity of the electrode enhanced to varying degrees caused by the polarization of the electrode materials. The 1Nb-FTO-S electrode provided a high initial specific capacity of 782.1 mAh g^−1^ at the rate of 50 mA g^−1^ thanks to some side reactions and the formation of the SEI film during the first discharge.

The CV curves of the 1Nb-FTO-S (1 mol% Nb) electrodes at voltages of 0–3.0 eV and a scan rate of 0.2 mV s^−1^ are provided in Figure 5c. The CV curves almost overlapped with each other, demonstrating the relatively stable electrochemical reversibility of the 1Nb-FTO-S electrode. The peaks of the first cycle cathodic reduction process are 0.13 and 0.66 V, respectively. The first peak indicates that Li is embedded inside the material, and this reaction may conceal the existence of an SEI film. The main reason for this embedding is related to the nature of the material. The XPS shows the coexistence of Fe^3+^ and Fe^2+^ in the sample due to the strong Jahn–Teller effect and the lengthening of the bond length between the Fe element and the oxygen element [38]. The octahedral distortion of the material [FeO_6_] is caused, which increases a large number of positions in the material lattice. At the same time, the material itself also has a high specific surface area, which is easy to adsorb Li atoms. In addition, due to the small adsorption barrier on the surface, it can be initiated when the voltage drop is very small, and the adsorption is reversible [8]. The second peak is located at 0.66 V, which is the redox reaction between the ferrous titanate and Li^+^. The peak in the oxidation process is between 1.11 and 1.89 V, which can be attributed to the oxidation of the Fe^2+^, Fe^0^, Fe^3+^, and Li^0^ in the material [9]. The EIS curves of the FTO-S, 1Nb-FTO-S, 5Nb-FTO-S, and 10Nb-FTO-S electrodes are presented in Figure 5d. The EIS spectra consisted of a semicircle and a sloping line. The semicircle in the high-frequency region was associated with the charge transfer reaction, and the straight line at low frequencies was ascribed to the solid-phase diffusion due to the Li-ion diffusion process in the nanosheets-based electrodes [38].

The charge transfer resistance (Rct) would reflect the ability of electrons and ions to transport inside the electrode. The Rct values of the FTO-S, 1Nb-FTO-S, 5Nb-FTO-S, and 10Nb-FTO-S electrodes were calculated as 18.5, 9.5, 24, and 64.5 Ω, respectively. The Rct values varied greatly among the different samples, and 1Nb-FTO-S (1 mol% Nb) displayed the lower Rct, indicating that Nb doping can lower the electrochemical polarization and improve the conductivity for a faster charge transfer capability within the electrode [39]. As shown in Table 1, a small amount of Nb doping enhanced the electrical conductivity of the samples, partly owing to charged ions of Nb^5+^ substituting transition-metal ions in the FTO that resulted in increased concentrations of electrons [40].

The cycling curves of the Nb-doping samples for 200 and 1000 cycles at the rates of 50 and 2000 mA g^−^^1^ are shown in Figure 5e and Figure 6f, respectively. At 50 mA g^−^^1^, the circulation capacity of the FTO-S sample looked relatively large. However, the specific capacity declined to 403 mAh g^−1^ after 200 cycles, corresponding to a loss of about 30%. 1Nb-FTO-S showed a high specific capacity and good cycle stability, with a specific capacity around 780.5 mAh g^−1^ for the first charge/discharge cycle and 537.8 mAh g^−1^ after three cycles. After 200 cycles, the specific capacity remained at 514.7 mAh g^−1^, equivalent to a capacity loss of about 4.3%. Thus, the 1Nb-FTO-S material showed an excellent cycling stability with a better lithium storage performance than those of many titanium-based ion cells anode materials, such as TiO_2_, Li_4_Ti_5_O_12_, and TiO_2_/FeTiO_3_ [8,10,11,33]. In Figure 5f, the sample volume decreased at a high rate of 2000 mA g^−1^, but the cycling stability remained good. The specific capacities of the FTO-S, 1Nb-FTO-S, 5Nb-FTO-S, and 10Nb-FTO-S samples after 1000 cycles were estimated to be 200.2, 220.6, 180.9, and 136.9 mAh g^−1^, respectively. Therefore, the samples possessed a superior cycle reversibility and cycle stability.

**Figure 7:**
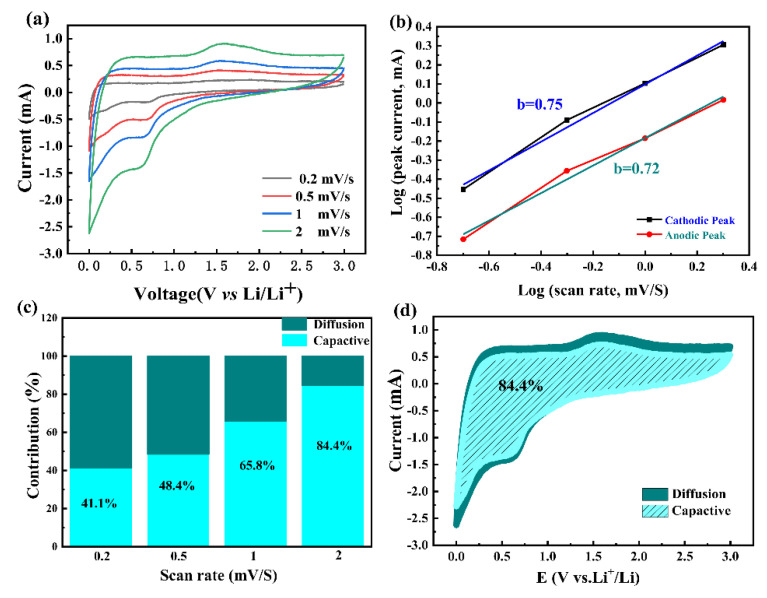
Electrochemical dynamics analysis: (**a**) CV curves of 1Nb-FTO-S anodes at various scan rates; (**b**) log(*i*) versus log(*v*) plots at different redox states, corresponding to CV curves in (**a**); (**c**) bar chart of pseudocapacitive/diffusion contribution of 1Nb-FTO-S anodes at different scan rates; (**d**) CV curves of 1Nb-FTO-S anodes with pseudocapacitive fractions at a scan rate of 2.0 mV s^−1^.

To further explain the electrochemical behaviors of the 1Nb-FTO-S (1 mol% Nb) electrodes, the CVs curves were recorded at different scanning rates of 0.2, 0.5, 1.0, and 2.0 mV s^−1^. As shown in Figure 7a, the current intensity increased with the scan rate. In terms of the electrode dynamics, the electrochemical reactions were divided into specific capacitance diffusive-controlled and capacitance-controlled processes, which can be determined by the relationship between the peak current (*i*) and scan rate (*v*) [41]:

log *i* = b log *v* + log a (1)

where a and b are adjustable parameters. Note that the b value represents the dominated charge storage behavior in the range of 0.5 to 1.0. A b value approaching 0.5 would indicate a diffusion-controlled redox process, while a b value approaching 1.0 would suggest a surface pseudocapacitive behavior process [42,43,44]. In Figure 7b, the b values at different redox states were calculated as 0.75 and 0.72, respectively. Hence, the reaction was governed by diffusion and pseudocapacitive co-contributing processes. The calculated capacitive contributions at the scan rates of 0.2, 0.5, 1.0, and 2.0 mV s^−1^ were estimated to be 41.1, 48.4, 65.8, and 84.4%, respectively (Figure 7c). The ratio of the capacitive contribution to the total capacity for the 1Nb-FTO-S electrodes is provided in Figure 7d. A value of 84.4% was recorded at the scan rate of 2.0 mV s^−1^.

Overall, the Nb-doped FeTiO_3_ nanosheets showed an excellent lithium-ion storage capability and good cycling stability due to their unique morphologies and microstructures. The performances of the electrode materials surpassed those of many other FeTiO_3_-based electrode materials (Table 3).

## 4. Conclusions

In summary, niobium-doping FeTiO_3_ nanosheets were successfully synthesized by the hydrothermal method. The substitution of Nb for Ti at the B-site induced the lattice distortion and the grain refinement, and the titania phase precipitates when the niobium-doping amount is large (5 and 10 mol%). Compared with the nanopowder, the nanosheet with a larger specific surface area has a better cycling performance and stability. A certain amount of Ti^3+^ and an oxygen vacancy can effectively improve the electrochemical performance of the electrode; a superfluous oxygen vacancy will lead to capacity attenuation. The conductivity of the electrode material increases first and then decreases with the increase in the Nb-doping amount. The highest conductivity of 1Nb-FTO-S (1 mol% Nb) obtained the best capacity and cycling performance, with the specific capacity of 782.1 mAh g^−1^ at 50 mA g^−1^. After 200 cycles, the specific capacity of the 1Nb-FTO-S electrode remained at 509.6 mAh g^−1^. Therefore, the as-prepared Nb-doping FeTiO_3_ nanosheets are considered to be a promising candidate for an LIB electrode material.

## Figures and Tables

**Table 1:** The structure parameters of Rietveld refinement.

Atom Type	Parameters	FTO	1Nb-FTO	5Nb-FTO	10Nb-FTO
**Fe**	***x***	0	0	0	0
	***y***	0	0	0	0
	***z***	0.3545(3)	0.3521(2)	0.3544(3)	0.3525(2)
	**100 ×** ***U_iso_*** **(Å^2^)**	1.51(4)	1.32(7)	0.76(6)	0.89(6)
	**Occupancy**	1.00	0.90(2)	0.89(2)	0.72(1)
**Ti/Nb**	***x***	0	0	0	0
	***y***	0	0	0	0
	***z***	0.1459(3)	0.1456(3)	0.1462(2)	0.1462(2)
	**100 ×** ***U_iso_(*** **Å^2^)**	0.65(4)	0.84(7)	1.33(7)	1.04(8)
	**Occupancy**	1.00	0.92(2)/0.01(1)	0.82(2)/0.02(1)	0.71(1)/0.05(1)
**O**	***x***	0.3206(6)	0.3242(7)	0.3184(6)	0.3126(6)
	***y***	0.0245(8)	0.0191(6)	0.0237(4)	−0.0032(2)
	***z***	0.2445(4)	0.2515(5)	0.2444(4)	0.2494(1)
	**100 ×** ***U_iso_*** **(Å^2^)**	1.41(6)	1.32(7)	1.21(8)	1.25(14)
	**Occupancy**	1.00	1.00	1.00	0.96(1)
**Reliability factors**	***R_wp_*** **(%)**	11.32	11.89	12.02	12.19
	***R_p_*** **(%)**	8.57	8.94	9.12	9.54
	***χ*** **^2^**	2.803	2.968	3.125	3.402

**Table 2:** Conductivity of Nb-doped samples (S cm^−1^).

Sample	Conductivity
FTO-S	2.1 × 10^−3^
1Nb-FTO-S	8.3 × 10^−2^
5Nb-FTO-S	2.5 × 10^−3^
10Nb-FTO-S	9.1 × 10^−3^

**Table 3:** The performances of reported FeTiO_3_-based electrode materials.

Research System	Discharge Capacity	References
Ilmenite FeTiO_3_ nanoflowers	400 mAh g^−1^ at 50 mA g^−1^ after 40 cycles	[8]
TiO_2_/FeTiO_3_@C fiber membrane	205 mAh g^−1^ at 300 mA g^−1^ after 100 cycles 150 mAh g^−1^ at 500 mA g^−1^ after 100 cycles	[10]
TiO_2_/FeTiO_3_@C	494.5 mAh g^−1^ at 100 mA g^−1^ after 150 cycles	[11]
TiO_2_/FeTiO_3_@C	441.5 mAh g^−1^ at 100 mA g^−1^ after 300 cycles	[45]
1%Nb-doped FeTiO_3_ nanosheets	514.7 mAh g^−1^ at 50 mA g^−1^ after 200 cycles 220.6 mAh g^−1^ at 2000 mA g^−1^ after 1000 cycles	This work

## Data Availability

Not applicable.
